# Breathing in Conversation

**DOI:** 10.3389/fpsyg.2020.575566

**Published:** 2020-10-14

**Authors:** Marcin Wlodarczak, Mattias Heldner

**Affiliations:** Department of Linguistics, Stockholm University, Stockholm, Sweden

**Keywords:** turn-taking, multiparty casual conversation, respiratory inductance plethysmography, breathing, interaction chronography

## Abstract

This work revisits the problem of breathing cues used for management of speaking turns in multiparty casual conversation. We propose a new categorization of turn-taking events which combines the criterion of speaker change with whether the original speaker inhales before producing the next talkspurt. We demonstrate that the latter criterion could be potentially used as a good proxy for pragmatic completeness of the previous utterance (and, by extension, of the interruptive character of the incoming speech). We also present evidence that breath holds are used in reaction to incoming talk rather than as a turn-holding cue. In addition to analysing dimensions which are routinely omitted in studies of interactional functions of breathing (exhalations, presence of overlapping speech, breath holds), the present study also looks at patterns of breath holds in silent breathing and shows that breath holds are sometimes produced toward the beginning (and toward the top) of silent exhalations, potentially indicating an abandoned intention to take the turn. We claim that the breathing signal can thus be successfully used for uncovering *hidden* turn-taking events, which are otherwise obscured by silence-based representations of interaction.

## 1. Introduction

The importance of breathing for production of speech needs little justification. It is, after all, the intricate coordinative patterns of the respiratory system that are the main driving force behind much of speech production as well as other vocal communicative behaviors. However, in spite of its importance, breathing has been generally overlooked in speech science. This claim can be easily verified by even a cursory look at standard phonetics textbooks with their focus firmly placed on articulatory phenomena and relatively little attention paid to the glottal and the subglottal systems. Supraglottal aspects of speech production enjoy a similar position of dominance when it comes to studies of communicative aspects of vocalizations in spontaneous conversation. While it is true that the field has enjoyed an increased interest in recent years, the contribution of the respiratory system to signaling speakers' communicative intentions is still far from clear.

In this paper, we present results on breathing turn-taking cues. Specifically, we study the respiratory patterns associated with initiating, holding, and releasing the turn. Unlike the previous studies (reviewed briefly in section 2.1), which focused primarily on properties of pre-speech inhalations, we investigate both inhalatory and exhalatory segments, as well as instances of respiratory holds. We also extend existing accounts by describing respiratory patterns found in overlapping speech. In addition, we present evidence that the respiratory signal can be used to identify turn-taking events which are otherwise obscured by the commonly used silence-based classification of conversational floor state (Jaffe and Feldstein, 1970). These include, above all, *pause interruptions*, which coincide with turn-holding silences in interlocutor's speech (Ferguson, 1977; Beattie, 1982; Gravano and Hirschberg, 2011). Since these instances involve no overlapping speech, they ostensibly resemble regular (smooth) speaker changes. We follow Shriberg et al. (2001) in referring to such occurrences as *hidden events*. This concept is further explained in section 2.2.

The study is based on two corpora of three-party spontaneous conversations in Swedish and Estonian. The analysis relies primarily on automatic methods for identification and parametrization of interactional and respiratory phenomena of interest, allowing for reproducible and comparable results across the data sets. The method is described in greater detail in section 3.

The results, presented in section 4, add a new aspect to the sizeable body of work on turn-taking cues in conversation (see e.g., Bögels and Torreira, 2015 for a recent review). They also contribute to the body of work on the role of respiratory cues in coordination and regulation of turn-taking (McFarland, 2001; Rochet-Capellan and Fuchs, 2014; Ishii et al., 2016; Włodarczak and Heldner, 2016b, 2018; Włodarczak et al., 2017) by including a wider range of interactional and respiratory phenomena. Additionally, in describing respiratory markers accompanying hidden turn-taking events, the study demonstrates how the respiratory signal might help overcome some of the deficiencies of using pause-delimited interactional units (Włodarczak and Wagner, 2013) by including speakers' unrealized intentions. Finally, given the latest developments in using the acoustic signal for tracking speech activity (Nallanthighal et al., 2019) as well as increasing availability of a wide range of sensors for remote tracking of breathing (Massaroni et al., 2018; Regev and Wulich, 2020), the findings can also inform models of turn-taking implemented in speech and interaction technology systems. We discuss these and other implications of the present work in section 5.

## 2. Previous Work

### 2.1. Respiratory Turn-Taking Cues

As indicated above, studies of respiratory mechanisms employed in production of spontaneous speech are rare. Even less frequent are studies of respiratory patterns underlying management and coordination of turn-taking. For instance, while Winkworth et al. (1995) characterized respiratory patterns in spontaneous speech, their study was predominantly concerned with variability of breath patterns, location of inhalations with respect to linguistic structure, and the influence of emotional state, rather than with turn-taking *per se*. Consequently, their material consisted of conversations with an experimenter “designed to maximize the number of subjects' utterances by providing appropriate questions and prompts” (p. 127) rather than eliciting natural turn-taking behavior. In addition, the conversations they investigated were relatively short (about 4 min) and involved subjects who were immobilized by means of shoulder straps, footrests, and hands held clasped on the lap.

By contrast, the first description of respiratory turn-taking cues proper was done by McFarland (2001). In this work, he examined duration patterns in breathing accompanying listening and speaking in dyadic situations, and compared them with quiet breathing (without any interlocutor present). The data included two dyadic situations: scripted dialogue (10 dyads, about 50 min) and spontaneous conversation (same 10 dyads, about 2 h 30 min), as well as quiet breathing (same 20 participants, about 40 min). With respect to the comparison of quiet breathing with the dyadic conditions, he found longer inhalations and shorter exhalations in the quiet breathing condition. Interestingly, he also noted a more speech-like respiratory pattern during listening than in quiet breathing (cf. Conrad and Schönle, 1979). With respect to the comparison of listening and speaking, he found a tendency to shorter inhalations in speaking than in listening, but this difference reached significance in the scripted dialogue condition only. Furthermore, he found a tendency to longer exhalations in speaking than in listening, but this difference was significant only in the spontaneous conversation condition. He also mentions a tendency toward longer exhalations in preparation for speaker change as well as longer exhalations in the first respiratory cycle following the speaker change. Thus, this work provides some support for longer exhalations and shorter inhalations (in potential next speakers) as a preparation of the respiratory system for speech production and speaker change and hence as turn-taking cues, but the results were not unambiguous.

Rochet-Capellan and Fuchs (2014) investigated the hypothesis that breathing “could be specifically involved in turn-taking and could constitute a coordinative unit for turn-exchange” (p. 3). To this end, they collected a series of short 2.5 min dyadic conversations between 11 participants and 2 confederates (for a total of about 4 h 35 min) and classified the turn-taking events according to a version of the scheme proposed by Gravano and Hirschberg (2011). This scheme included characterizing pause-delimited utterances as either backchannels, turn-holding or turn-taking. The turn-taking category was further subdivided into (1) non-competitive *smooth transitions* occurring after complete turns, (2) competitive *interruptions*, in which the incoming speaker successfully grabs the floor from the interlocutor, and (3) competitive *butting-ins*, in which the incoming speaker fails to interrupt the interlocutor^1^. They identified onsets of inhalations and exhalations automatically using velocity criteria and corrected them manually when needed. From these respiratory events, they calculated inhalation, exhalation and breathing cycle durations, breathing cycle asymmetries, and breathing rates, as well as the temporal alignment of the inhalation onset to the respiratory cycle of the other speaker. Furthermore, by combining the respiratory events with the utterance segmentations, they calculated the position of the speech onset in relation to the exhalation phase. They found most turns to be completed within one breathing cycle and almost all in fewer than four. As expected, the great majority of turns were initiated early in the exhalation (over 50% of cases fall within the initial 25% of its duration), with butting-ins occurring generally later. They also identified differences regarding durational properties, with turn-holding being characterized by shorter respiratory cycles than turn-taking, and showed that this was predominantly due to a reduction in inhalation duration. By contrast, visual inspection of their **Figure 4** suggests that inhalation durations in turn-taking are comparable to those in listening (cf. McFarland, 2001). Furthermore, butting-ins resulted in shorter cycles than smooth turns and interruptions were more systematically coordinated with the end of the interlocutor's exhalation phase than smooth transitions. However, these observations are likely side effects of the premature termination of the incoming turn (for butting-ins) or the previous turn (for interruptions) rather than planning on the part of the incoming speaker. Thus, this work provides support for shorter inhalations (in current speakers) as turn-holding cues. However, it did not provide any definite evidence for breathing profiles differentiating competitive from non-competitive turn-takings.

In further analyses of the same material, Rochet-Capellan et al. (2014) noted a tendency that the shorter inhalations in turn-holding were also accompanied by shorter silent intervals before as well as after the inhalation (i.e., between offset of speech and onset of inhalation, and between offset of inhalation and onset of speech). Thus, the entire “breath pause” was temporally compressed in turn-holding.

Within a more constrained domain of question and answer sequences (*N* = 171) in Dutch, Torreira et al. (2015) reported that almost 47% of answers were not preceded by an inhalation (i.e., the inhalation occurred before the question onset), however, pre-speech inhalations were more common before longer answers than before shorter ones. Additionally, answers preceded by an inhalation were delayed with respect to the previous utterance to a greater extent than answers produced on residual air, suggesting that the latter strategy might be employed to avoid long between-speaker silences. When present, the inhalations started most commonly shortly (15 ms) following the question offset, although a large variation was present. The results were, thus, consistent with the utterance planning model, according to which planning of the next utterance starts early but is triggered by turn-final yielding cues (“go-signal”) (Levinson and Torreira, 2015; Barthel et al., 2016, 2017).

All the studies referred to above studied turn-taking in dyadic conversations, which can be thought of as the simplest form of turn negotiation. Indeed, a scenario involving two participants leaves open only the question of *when* speaker change is going to occur, and not *which* of the several competitors for the turn is going to claim it. By contrast, turn competition between two or more potential next-speakers in multiparty conversation is likely to result in more complicated turn-taking patterns, which, in turn, might be reflected in interlocutors' breathing behavior. This possibility was investigated by Ishii et al. (2016), who recorded respiratory activity in eight spontaneous four-party conversations (for a total of 1 h 36 min) and looked for patterns signaling turn-holding, turn-yielding as well as an intention to initiate a new turn. Utterances, turns and turn-taking events were segmented and classified manually. Intervals of overlapping speech as well as backchannel-like “supportive responses” were excluded from the analyzed material. The respiratory features described the inhalations only. Onsets and offsets of inhalations were identified automatically using the sign of the derivative of a low-pass filtered respiratory signal (however, at the cost of a substantial data loss). Inhalation duration, amplitude, and slope as well as timing relative to one's own preceding and following speech were estimated using these landmarks.

The paper reported a great number of comparisons, only some of which are relevant and meaningful for modeling of turn-taking mechanisms. Overall, post-speech inhalation amplitude was found to be larger in turn-holding than in turn-yielding. The authors also observed the temporal compression reported previously by Rochet-Capellan et al. (2014) and Rochet-Capellan and Fuchs (2014) during turn-holding, that is, a generally shorter “breath pause” between utterances in turn-holding. With respect to respiratory markers of claiming the turn, results were weak. There were no differences in inhalation duration in next speakers, and only marginally larger amplitudes. The authors conclude the paper by proposing a three-step prediction model which at every pause-delimited utterance offset: (1) discriminates between turn-keeping and turn-yielding, (2) in the latter case, predicts the next speaker, and (3) predicts the silence duration. The model was demonstrated to improve on the baseline model based on average silence duration in turn-keeping and turn-yielding.

A more realistic method of evaluating the relative contribution of the respiratory signal to prediction of speech activity in multiparty dialogue was used by Włodarczak et al. (2017). Instead of discriminating between turn-keeping and turn-yielding at utterance offset, their model predicted whether or not a particular participant will be speaking within the next 100-ms window, based on this participant's 1-s speech activity and respiratory history. The results showed that respiratory information improved prediction of incipient speech activity, compared to a baseline model trained on speech activity alone (rather than a simple majority class, as was done in Ishii et al., 2016). By contrast, interlocutors' respiratory patterns offered no predictive advantage, which suggests that breathing history of a conversation participant is only helpful for predicting this participant's future vocalization but not that of their conversational partners. In other words, conversational partners do not seem to orient to each other's breathing for effecting speaker change. In addition, *z*-normalized respiratory slope within a 100-ms window was found to be the best performing feature among those compared.

As should be apparent from the above overview, even though studies of breathing and turn-taking have been rather rare they differ widely with respect to the amount and type of data used (scripted dialogues, short interactions with confederates, question-answer pairs, multiparty conversations), data acquisition choices (one or two belts) and the aspects of the respiratory signal selected for analysis. These differences notwithstanding, the studies provide evidence for systematic variation in breathing in the vicinity of turn-taking events, with the shortening of the inhalation in turn-holding being the most robust cue found. By contrast, breathing patterns related to claiming or releasing the turn were less consistent. Even though the effects were generally weak, the breathing signal was demonstrated to improve prediction of turn-taking in technical systems.

### 2.2. Classification of Turn-Taking Events

In order to characterize turn-taking events, they first have to be identified as such. Several approaches to the classification of turn-taking events have been used in the past. The speaking turn, as introduced in the seminal work by Sacks et al. (1974), is defined in terms of projectable units whose syntactic, semantic and prosodic completeness can be inferred from the ongoing stream of speech. These predictable completion points (or transition-relevance places, in Conversation Analysis speak) are locations where speaker change can occur according to a set of rules whereby the previous speaker selects the next, the next speaker self-selects or, barring other turn-contestants, the previous speaker continues. Conceived of in this way, the turn consists of prosodically, syntactically and pragmatically complete units which need to be identifiable in a reliable and time-efficient way if Sacks et al.'s system is to be applied to the task of corpus segmentation. These aspects become particularly crucial in large-scale corpus studies.

**Figure 1:**
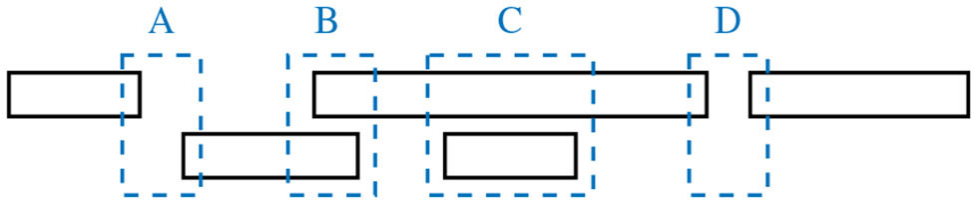
Turn-taking categories: between-speaker silence, BSS **(A)**; between-speaker overlap, BSO **(B)**; within-speaker overlap, WSO **(C)**; and within-speaker silence, WSS **(D)**. The top and bottom boxes represent individual speakers' vocalizations.

An alternative approach to dialogue segmentation rests on interactional units identified in a fully mechanistic fashion based on the notion of *speaker change*. This technique goes back to the method of interactional chronography, introduced by Norwine and Murphy (1938) and developed further by Brady (1968) and Jaffe and Feldstein (1970). Briefly, the method consists of identifying talkspurts or interpausal units, that is intervals of speech (or voice activity) delimited by pauses longer than a predefined threshold^2^. The possible turn configurations are then defined by application of two criteria: (i) presence of overlapping speech (contrasting silences and overlaps), (ii) presence of speaker change (contrasting between- and within-speaker intervals). The resulting system thus comprises four categories, depicted schematically in Figure 1: within-speaker silence (WSS), within-speaker overlap (WSO), between-speaker silence (BSS), and between-speaker overlap (BSO).

Interactional chronography has clear benefits: it is automatic, fully reproducible and efficient. However, since it also decouples the task of turn segmentation from the notion of speakers' intentions, it is unable to distinguish between *competitive* and *non-competitive* (or problematic and non-problematic) speaker changes (Schegloff, 2000, 2001). For instance, it cannot distinguish *pause interruptions*, in which the incoming speaker starts speaking during what was intended as a turn-holding pause by the previous speaker, from regular *smooth speaker switches*. In a chronogram they look the same. Even though such distinctions have been incorporated into turn-taking labeling schemes, notably in the scheme initially proposed by Ferguson (1977) which was later adapted first by Beattie (1982) and then by Gravano and Hirschberg (2011), and used in corpus studies (Gravano and Hirschberg, 2011; Rochet-Capellan and Fuchs, 2014), their identification requires manual annotation. In this paper, we follow Shriberg et al. (2001) in referring to such interactional events obscured by a particular representation of the phenomenon under study as *hidden events*.

While pause interruptions are the archetypal example of a hidden turn-taking event, they are by no means the only one. It is the same situation with speaker switches involving overlapping speech, where non-competitive *overlaps* and competitive *interruptions* (Gravano and Hirschberg, 2011) cannot be distinguished based on chronograms. Furthermore, a speaker might want to release the turn but nevertheless find herself having to continue in the face of no other turn contestants. Similarly, a potential speaker might be getting ready to start a turn but might be prevented from speaking by a faster interlocutor. However, these eventualities have so far received little attention in turn-taking literature, not least because of the difficulties in identifying them reliably.

In our earlier work (Włodarczak and Heldner, 2018), we analyzed kinematic properties of post-speech breathing patterns and found a number of between-speaker silence intervals in which the first speaker produced more speech after the second speaker's utterance without making an inhalation. We hypothesized that these cases correspond to instances of pause interruptions and demonstrated that the respiratory characteristics of these intervals was consistent with this idea. Namely, we showed that these intervals have similar respiratory characteristics to turn-holding silences, uninterrupted by another speaker. Specifically, they have a less steep slope, are started higher in speaker's respiratory range and are longer than exhalations accompanied by a speaker change. In fact, exhalations coinciding with these pause interruptions were the longest of all the categories we investigated, suggesting that the previous speaker might be holding their breath while waiting for the incoming speech to end.

To the best of our knowledge, the only other respiratory study which considered interruptions as a separate category was Rochet-Capellan and Fuchs (2014). In that work, the authors differentiated between *smooth turns*, successful *interruptions* and failed *butting-ins*, and found that butting-ins were associated with shorter and less asymmetrical respiratory cycles. They also occurred later in the exhalatory phase than the other two categories. Notably, the analysis was based on manual classification of turn onset types and the analyzed classes pooled speaker changes accompanied by silence and overlap.

In this paper, we revisit the idea of pause interruptions from Włodarczak and Heldner (2018) using a larger multilingual data set, comprising Swedish and Estonian conversational material. Unlike Rochet-Capellan and Fuchs (2014), our analysis does not rely on manual labels of interruptions. Instead, we try to identify interruptions based on respiratory features of selected interactional events. Because we are primarily interested in whether the previous speaker has yielded the floor, we focus on features of the post-speech exhalatory segment rather than the inhalation preceding the incoming talkspurt. We also extend the analysis in Włodarczak and Heldner (2018) in several ways: (i) we include an automatic annotation of breath holds, and (ii) we analyse respiratory patterns of between-speaker overlaps and demonstrate that they also show the expected pattern of interruption. In addition, we propose a method for identifying the abandoned intention to take the floor, where the speaker was planning to initiate a turn but produced no speech. Namely, we identify silent cycles which involve a respiratory hold in the top portion of the exhalatory phase.

## 3. Method

In total, 18 three-party conversations were used in the study: 8 in Swedish and 10 in Estonian. All subjects were native speakers of the respective languages and, with the exception of a single Swedish conversation, knew each other prior to the recording. The subjects were instructed to engage in a casual conversation on a topic of their choice for about 20 min. All conversations were recorded using an identical setup in the Phonetics Laboratory at the Department of Linguistics, Stockholm University. The subjects were recorded standing at a round bar table (105 cm in height) to minimize distortions in the respiratory signal. Speech was recorded using directional close-talking condenser microphones (Sennheiser HSP 4) to reduce the amount of cross-talk.

**Figure 2:**
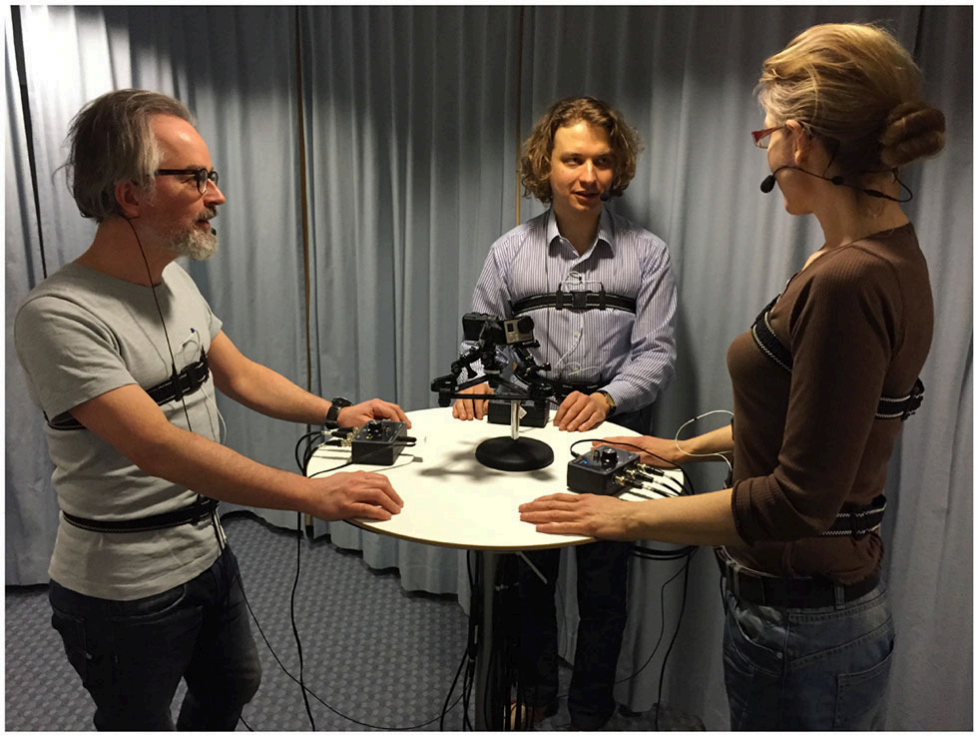
Recording setup in the Stockholm University Respiratory Lab.

Respiratory activity was measured using the Respiratory Inductance Plethysmography (RIP) method (Cohn et al., 1978; Watson, 1980), as implemented in the RespTrack system developed in the Phonetics Laboratory at Stockholm University (Heldner et al., 2019). Very briefly, the RIP method uses two elastic inductive belts worn around the chest and the abdomen to trace respiratory movements (see Figure 2). Inhalations and exhalations alter the circumference as well as the inductance of the belts. The belts are connected to electronics that convert the varying inductance into (analog) direct current (DC) signals with amplitudes that are approximately proportional to the changes in circumference. In addition to the individual signals from the rib cage and abdomen belts, the RespTrack system provides a weighted sum of these signals, allowing a direct estimation of lung volume change. Correct weighting is obtained by instructing the subject to close the glottis, and then to repeatedly contract and relax the abdominal wall, while the experimenter adjusts a potentiometer knob on the RespTrack main unit so that the summed signal remains flat when air is moved from the abdomen to the chest. This is the so called *isovolume manoeuvre* (Konno and Mead, 1967). A slightly higher weight to the rib cage belt is usually required (Banzett et al., 1995). An important feature of the RespTrack system given the objectives of this study is the method used for correcting DC offset in the belt signals. Unlike many other RIP systems, RespTrack does not use high-pass filtering for this purpose, which permits distinguishing breath holds from periods of slow exhalations. The RIP as well as the microphone signals were digitized with an integrated data acquisition system (PowerLab 1635 hardware and LabChart software from AD Instruments).

Talkspurts were identified automatically, using the voice activity method described in Laskowski (2011) with the standard 100-ms frame and time step. The minimum pause duration between two adjacent talkspurts was set at 200 ms. Subsequently, interaction chronograms (including turn-taking events) were created using TextGridTools, a Python toolkit for working with Praat TextGrid files (Buschmeier and Włodarczak, 2013). Intervals of laughter were identified using the method by Ryokai et al. (2018) with the code and models accompanying the paper^3^. The authors report a per-frame accuracy of 88% on a held-out Switchboard test set, which is comparable to state-of-the-art performance of audio-only automatic laughter recognizers (Cosentino et al., 2016).

The respiratory signal was processed using RespInPeace, a Python toolkit for analysing RIP data (Włodarczak, 2019). Specifically, segmentation into inhalatory and exhalatory segments was done by locating peaks and troughs in the *z*-scored respiratory signal separated by at least one standard deviation^4^. Similar to our previous work (e.g., Włodarczak and Heldner, 2016b), each participant's lung volume used for speaking (henceforth, *speaking volume*, SV) was calculated as the interval between the 5th and 95th percentiles of all peak and trough values, and resting expiratory level (REL) was estimated dynamically as the median value of troughs within a 60-s window. In addition, breath holds were identified using the method proposed by Noto et al. (2018) for airflow recordings, adapted to the RIP signal and also included in the RespInPeace toolkit. Briefly, since a respiratory hold shows up as a plateau in the RIP signal, the method looks for prominent peaks in histograms of the RIP signal values in each breathing cycle and then identifies the time interval when the signal stays within some margin around the peak. In addition we set the minimum hold duration to 250 ms and the minimum gap between two holds to 150 ms. Given that the method often mistakes speech segments, which also produce slowly decaying regions approximating plateaus, for breath holds, only hold candidates produced during periods of silent exhalations were included. Noto et al. (2018) evaluated the original breath hold detection method using simulated data, the 95% confidence intervals for average breath hold duration coinciding with inhalations or exhalations were very narrow, spanning between 0.006 and 0.02 ms.

**Figure 3:**
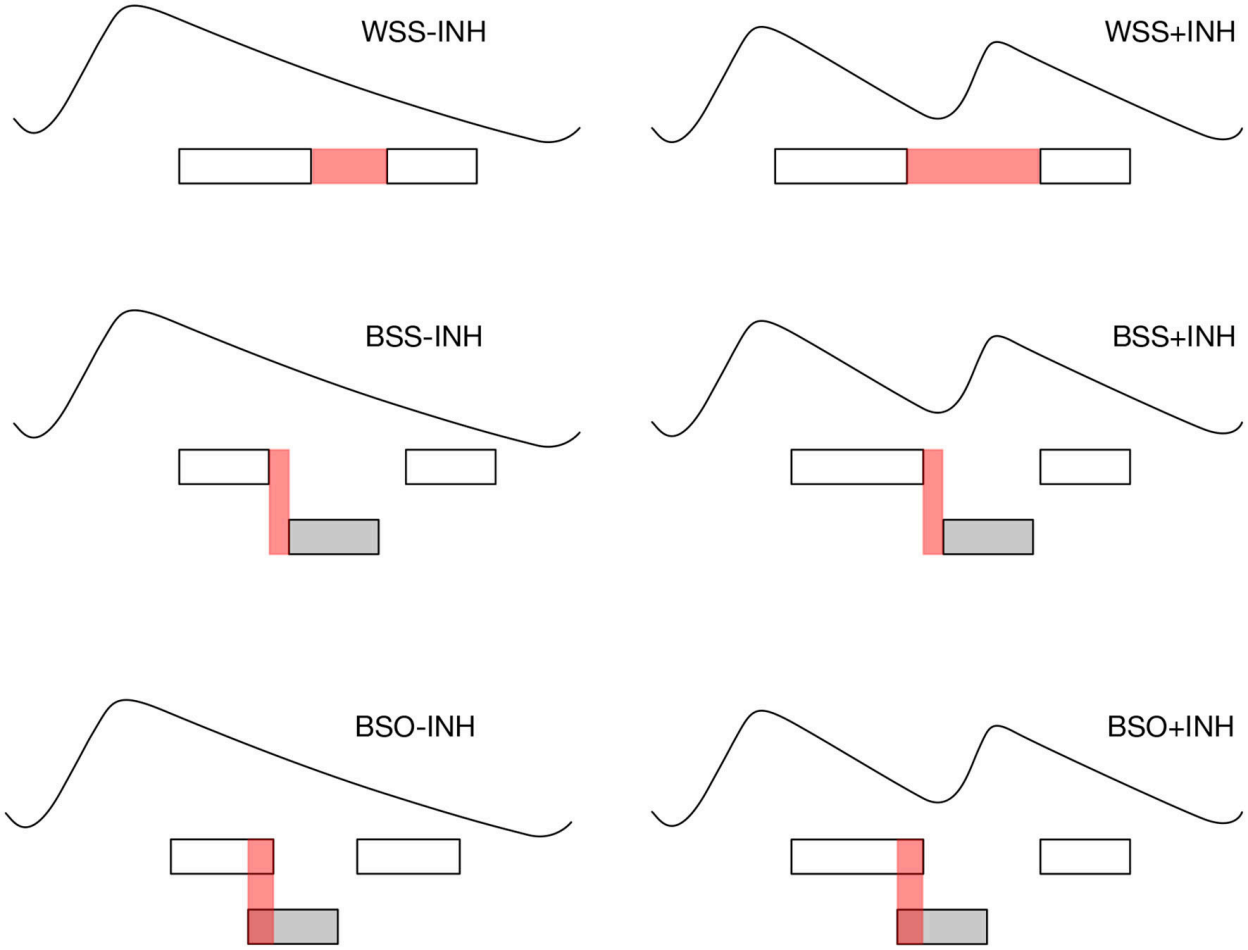
Interactional intervals classified with respect to presence of speaker change, overlapping speech and inhalation in the previous speaker's respiratory pattern.

For the purpose of the present study, we further classified the WSS, BSS, and BSO intervals depending on whether the following utterance by the original speaker was preceded by an inhalation (+INH) or directly followed the exhalatory segment (-INH), see Figure 3 for an illustration. For the category not involving speaker change (WSS) it is simply a matter of whether the speaker inhales before continuing. For the categories involving speaker change (BSO and BSS), we search for the next utterance by the original speaker and check whether it is preceded by an inhalation. Thus, for instance, the WSS-INH class comprises those instances of within-speaker silences in which the speaker continues without breathing in and the BSS+INH class includes between-speaker silences in which the original speaker inhales before producing her next talkspurt. Given that our automatic method of segmenting the respiratory signal sometimes misses small inhalatory segments, possibly inflating exhalation durations of the BSO-INH and BSS-INH intervals^5^, we manually checked whether there was, in fact, no inhalation present. Cases of missed inhalations (*N* = 73) were excluded from the analysis. Also excluded were all intervals in which edges of the speech segments coincided with an inhalation (*N* = 4, 521)^6^ or which included laughter (*N* = 1, 801).

**Figure 4:**
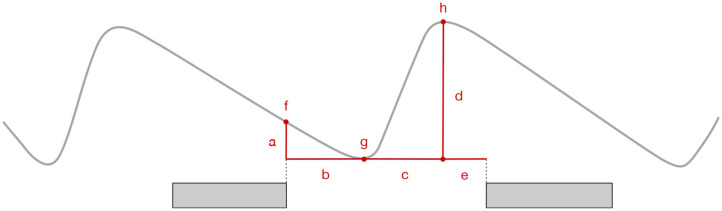
Respiratory features: (a) exhalation amplitude, (b) exhalation duration, (c) inhalation duration, (d) inhalation amplitude, (e) speech lag, (f) exhalation onset level, (g) exhalation offset/inhalation onset level, (h) inhalation offset level. Exhaltory and inhalatory slopes were calculated as a/b and d/e, respectively. The gray bars represent talkspurts.

For the remaining intervals, we extracted amplitude (expressed in units of SV), duration (in log_2_ s), slope (in SV per second), speech lag (the duration between the inhalation onset and speech onset, in log_2_ s^7^), and lung volume (as fractions of SV) at the onset and the offset of the exhalatory segment following the previous speaker's talkspurt (for WSS intervals, we took the exhalation following the pre-pausal talkspurt). In addition, for [+INH] intervals, we extracted features of the inhalation (duration, amplitude, slope, and offset level) preceding the next talkspurt of the previous speaker. For between-speaker intervals, the same features were extracted from the inhalation preceding the talkspurt produced by the next (incoming) speaker, further classified according to whether the between-speaker interval involved a silence, TT(S), or an overlap, TT(O). The measures are illustrated in Figure 4.

Since backchannels are generally assumed not to claim the conversational floor (Yngve, 1970), all the turn configurations (both between- and within-speaker) involving at least one backchannel were excluded from the analysis (*N* = 5, 055). Also excluded were inhalations and exhalations with extreme values of slope (at least three standard deviations away from the mean, *N* = 94). In this work, we operationalized backchannels as talkspurts shorter than 1 s. This criterion, proposed by Heldner et al. (2011) was previously demonstrated to be an accurate proxy for the backchanel/non-backchannel distinction. The frequencies of the analyzed intervals are listed, separately for inhalations and exhalation, in Table 1 alongside the percentage of instances in each category coinciding with a respiratory hold.

**Table 1:** Frequencies of the analyzed turn-taking categories, alongside the percentage of instances in each category coinciding with a respiratory hold.

	**Exhalations**		
**Category**	**Frequency**	**% holds**	**Inhalations**
WSS-INH	578	2	—
WSS+INH	230	1	248
BSS-INH	35	10	—
BSS+INH	461	6	495
BSO-INH	35	8	—
BSO+INH	207	3	216
TT(S)	—	—	441
TT(O)	—	—	203

*See section 3 for explanation of the categories*.

Additionally, we identified abandoned speech candidates as silent cycles accompanied by a respiratory hold occurring in the top 20% of the exhalation amplitude. In other words, these are the cases where a conversation participant holds their breath right at the beginning of an exhalation which does not coincide with their own speech^8^. For these intervals we extracted inhalation duration and amplitude, and compared them against silent cycles without respiratory holds. This procedure identified 221 abandoned initiation candidates, which were compared against 6,121 silent cycles not coinciding with respiratory holds.

The inhalatory and exhalatory features were modeled separately using multinomial logistic regression in R, using the *mlogit* package. Models were built step-wise by adding one predictor at a time and checking whether including the predictor significantly reduces −2 × log-likelihood of the resulting model.

The breathing data as well as the code used for feature extraction, preprocessing as well as statistical analysis is available online at https://doi.org/10.5281/zenodo.4054803.

## 4. Results

This section gives an overview of some of the breathing patterns related to floor management in multiparty casual conversation. We start with breath holds and exhalatory features, which have received very little attention to date, continue onto the more familiar ground of inhalatory properties and conclude with the rather peculiar phenomenon of breath holds found in the middle of silent breathing cycles.

### 4.1. Breath Holds in Turn-Taking

Co-occurrence of respiratory holds with the analyzed turn-taking categories is shown in Table 2. Notably, only 1–2% of WSS intervals involved a respiratory hold, suggesting that respiratory holds are not routinely employed for maintaining possession of the conversation floor. Conversely, the category with the highest likelihood of coinciding with a respiratory hold was BSS-INH (10%), which, as we indicated above, is likely to involve some degree of turn-competition. The same is at least partly true for its overlapped counterpart, BSO-INH, which coincided with a respiratory hold in 8% of cases.

**Table 2:** Coefficients of the exhalatory model.

				**95% CI**		
		**B**	**exp(B)**	**LL**	**UL**	***p***
BSO+INH	Intercept	−1.65	0.19	−1.96	−1.33	0.00
	Offset level	−11.12	0.00	−12.69	−9.55	0.00
	Slope	−9.93	0.00	−11.74	−8.11	0.00
	Hold = True	1.04	2.84	−0.03	2.11	0.06
BSO-INH	Intercept	−2.84	0.06	−3.40	−2.29	0.00
	Offset level	−0.81	0.44	−2.73	1.10	0.40
	Slope	−1.85	0.16	−5.09	1.38	0.26
	Hold = True	1.70	5.45	0.34	3.05	0.01
BSS+INH	Intercept	−0.65	0.52	−0.90	−0.40	0.00
	Offset level	−11.82	0.00	−13.20	−10.44	0.00
	Slope	−8.66	0.00	−10.28	−7.04	0.00
	Hold = True	1.58	4.84	0.73	2.43	0.00
BSS-INH	Intercept	−2.81	0.06	−3.36	−2.26	0.00
	Offset level	−0.94	0.39	−2.87	0.99	0.34
	Slope	−1.70	0.18	−4.93	1.53	0.30
	Hold = True	1.97	7.19	0.74	3.21	0.00
WSS+INH	Intercept	−1.49	0.22	−1.80	−1.19	0.00
	Offset level	−9.16	0.00	−10.64	−7.68	0.00
	Slope	−9.75	0.00	−11.51	−8.00	0.00
	Hold = True	−0.37	0.69	−1.95	1.21	0.64

*The reference category is WSS-INH*.

*R^2^ = 0.18 (McFadden), model χ^2^_(20)_ = 826.54, p < 0.01*.

In order to verify to what extent breath holds are used proactively and to what extent they are produced to ward off (or wait out) an interlocutor's interruption, we calculated conditional probabilities of speaker change depending on whether or not a respiratory hold is present. If respiratory holds were used as an effective turn-holding signal, the probability of a speaker change in their presence, *P*(speaker change|hold), should be low compared to the probability of a speaker change not accompanied by a hold, *P*(speaker change|no hold). Conversely, if they are used in response to interlocutor's interruption, the opposite should be true. We find that the probability of a speaker change is much higher (0.74) when a silence coincides with a respiratory hold than when it does not (0.38), suggesting that holds are produced predominantly reactively, in response to incoming speech.

### 4.2. Exhalatory Features

**Figure 5:**
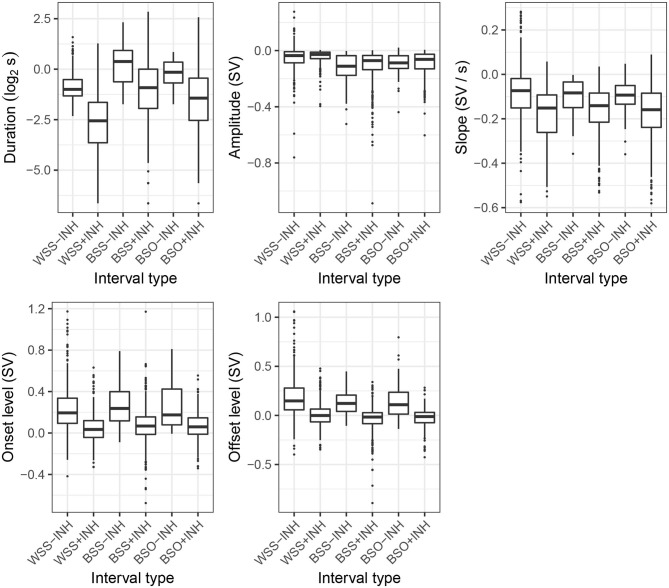
Distributions of exhalatory features across the interval types. See section 3 for explanation of the categories.

Distributions of exhalation duration, amplitude, and slope as well as onset and offset levels in the six interval types are plotted in Figure 5. For all features, with the exception of amplitude, the categories form two separate groupings for intervals accompanied with an inhalation [+INH] and intervals in which the previous speaker's next talkspurt directly follows the exhalatory segment [−INH]. Specifically, exhalations in the latter category start and end higher in the respiratory range (and generally above REL, corresponding to 0 on the ordinate), unlike the [+INH] intervals which, predictably, end in the vicinity of REL. The [−INH] intervals are also characterized by less steep slopes. Notably, the fact that the distributions of these features for BSO-INH and BSS-INH are similar to the turn-holding WSS-INH suggests that the presence of speaker change in the absence of an inhalation might indicate that the previous speaker did not intend to release the turn and the incoming speech is of an interruptive character.

The distributions of exhalatory duration show a similar grouping with [−INH] intervals being generally longer than their [+INH] counterparts. In addition, several effects previously attested in the literature are also apparent in that plot. For instance, as observed in several studies (Rochet-Capellan and Fuchs, 2014; Ishii et al., 2016; Włodarczak and Heldner, 2016b), the short exhalations in WSS+INH are a strong correlate of turn-holding. What is more interesting in the context of the present study is that the between-speaker [−INH] intervals (i.e., BSO-INH and BSS-INH) show the longest durations of all the categories, followed by BSO+INH. We interpret this finding as evidence of turn-competition at these junctures in the conversations and come back to this point in the Discussion below.

By contrast, exhalation amplitude shows a different grouping where within-speaker intervals, whether or not accompanied by an inhalation, involve a shallower exhalation than the other interval types.

The contribution of individual features to prediction of interval type was assessed using multinomial logistic regression. Due to high collinearity between duration, amplitude and slope, only slope was used as composite feature. Onset and offset levels were also highly correlated; consequently, only the latter was used as a predictor. All the resulting predictors (slope, offset level, presence of respiratory holds) significantly improved fit of the resulting model (indicated with likelihood ratio test) and were included in the final model, summarized in Table 2. WSS-INH was used as the reference category due to of its unambiguously turn-holding character.

**Figure 6:**
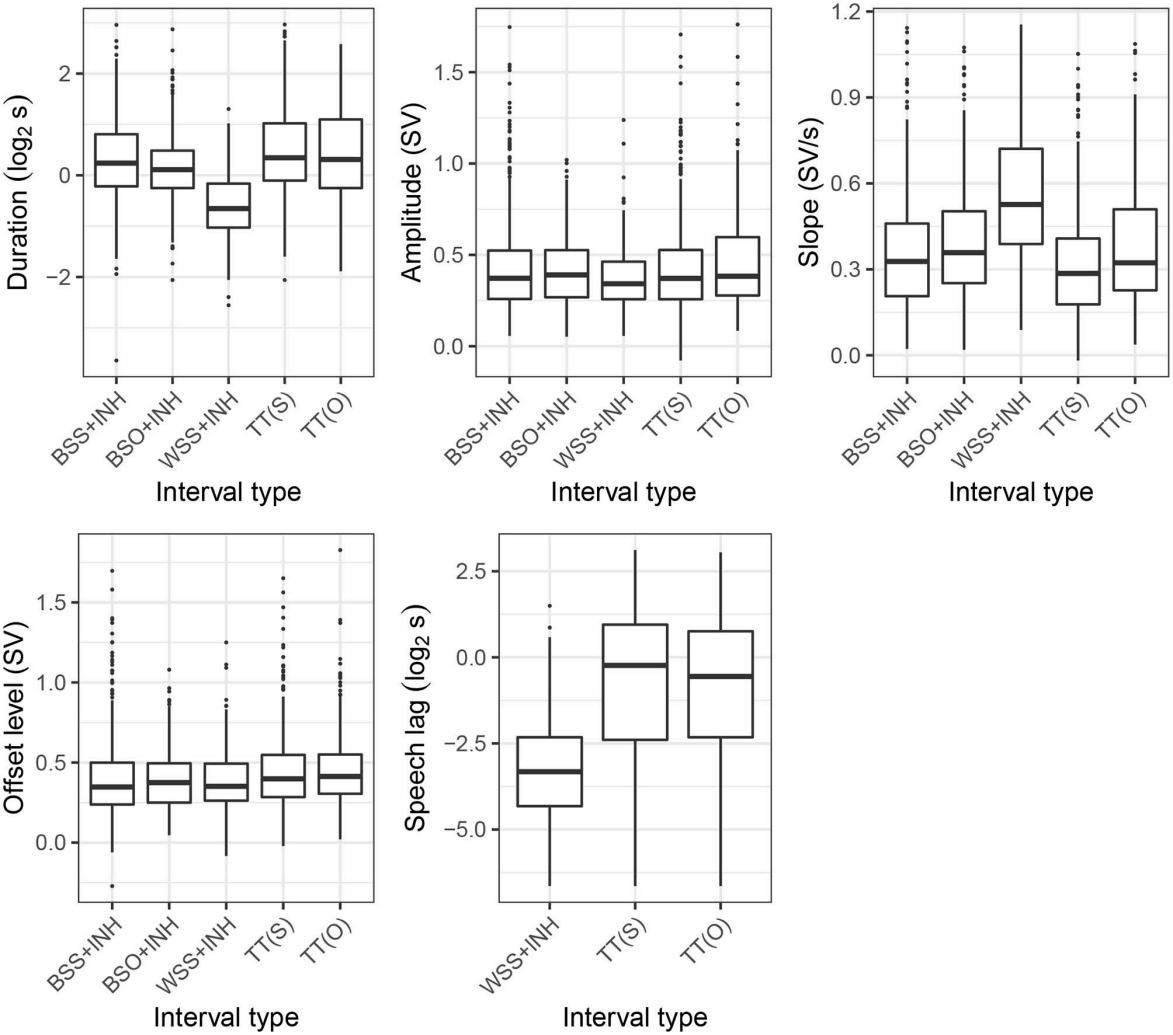
Distribution of inhalatory features across the interval types. See section 3 for explanation of the categories.

The results of the logistic regression are largely in line with the two-way grouping suggested in Figure 6. Neither BSO-INH nor BSS-INH are significantly different from the WSS-INH category as far as slope and onset level are concerned. By contrast, all [+INH] categories are significantly different from the reference, whereby higher offset levels and higher (less negative) slope values reduce the odds of the [+INH] classes. Presence of respiratory holds was a significant predictor for all categories, except for WSS+INH and (marginally) BSO+INH. In all other cases, respiratory holds increase the odds of the predicted category compared to the reference. McFadden's pseudo-*R*^2^ for this model equals 0.18; if the duration and onset level are also included in the model^9^, the value goes up to 0.25, indicating very good model fit, with values between 0.2 and 0.4 being equivalent to *R*^2^ values of 0.7–0.9 for linear functions (McFadden, 1978; Louviere et al., 2000).

### 4.3. Inhalatory Features

We now turn to properties of the inhalations. Figure 6 presents distributions of five inhalatory features: amplitude, duration, slope, offset level, and speech lag. Obviously, these features were only calculated for the [+INH] as well as the turn-taking intervals, TT(S), TT(O). Speech lag was only calculated for WSS+INH, TT(S), and TT(O). Onset level was not included since it is equal to exhalation offset level (see Figure 4).

Overall, there was very little amplitude and offset level variation across the five categories. In particular, there was no marked difference between the turn-releasing BSO intervals and the TT intervals. We return to this point in the discussion.

The distributions of slope, duration, and speech lag show primarily the familiar temporal compression pattern attested previously in literature: turn-holding WSS intervals are characterized by steeper (more positive) and shorter inhalations, which are followed much more quickly by speech. Again there is no substantial difference between BSO and TT intervals, and only slightly steeper slopes in intervals involving overlap, BSO+INH and TT(O), than in those coinciding with silence, BSS+INH and TT(S).

The contribution of the inhalatory features to prediction of interval type was assessed again using multinomial logistic regression. Similar to exhalatory features, only slope was included in the model due to high collinearity between slope, amplitude, and duration. Given that speech lag was defined only for a subset of intervals, we fitted two separate models. First, we fitted a model which included slope and offset level using all five interval types. Next, for WSS+INH, TT(S), and TT(O) intervals, we fitted a model which additionally included speech lag as a predictor. The results of these models are summarized in Tables 3, 4. In both models, WSS+INH was used as a reference.

**Table 3:** Coefficients of the inhalatory model for all intervals involving an inhalation.

				**95% CI**		
		**B**	**exp(B)**	**LL**	**UL**	***p***
BSO+INH	Intercept	0.72	2.05	0.25	1.19	0.00
	Offset level	1.98	7.26	0.93	3.04	0.00
	Slope	−3.45	0.03	−4.36	−2.54	0.00
BSS+INH	Intercept	1.76	5.84	1.35	2.18	0.00
	Offset level	2.68	14.56	1.75	3.61	0.00
	Slope	−4.77	0.01	−5.59	−3.96	0.00
TT(O)	Intercept	0.40	1.49	−0.08	0.87	0.11
	Offset level	3.83	45.99	2.81	4.85	0.00
	Slope	−4.82	0.01	−5.82	−3.82	0.00
TT(S)	Intercept	1.65	5.19	1.22	2.07	0.00
	Offset level	4.19	66.08	3.24	5.14	0.00
	Slope	−6.67	0.00	−7.60	−5.74	0.00

*The reference category is WSS+INH*.

*R^2^ = 0.06 (McFadden), model χ^2^_(12)_ = 297.43, p < 0.01*.

**Table 4:** Coefficients of the inhalatory model for WSS+INH, TT(O), and TT(S) intervals.

				**95% CI**		
		**B**	**exp(B)**	**LL**	**UL**	***p***
TT(O)	Intercept	1.43	4.18	0.83	2.03	0.00
	Offset level	3.03	20.73	1.86	4.21	0.00
	Slope	−3.85	0.02	−4.98	−2.71	0.00
	Speech lag	0.54	1.71	0.42	0.66	0.00
TT(S)	Intercept	2.61	13.55	2.04	3.17	0.00
	Offset level	3.41	30.18	2.30	4.51	0.00
	Slope	−5.53	0.00	−6.61	−4.46	0.00
	Speech lag	0.52	1.69	0.42	0.63	0.00

*R^2^ = 0.21 (McFadden), model χ^2^_(8)_ = 389.75, p < 0.01*.

*Unlike the model in Table 3, this model includes speech lag as a predictor, which is undefined for the other intervals. The reference category is WSS+INH*.

In the first model (Table 3), fitted on the whole data set, an increase in offset level (i.e., completing the inhalation higher in the respiratory range) is associated with higher odds of the predicted category against the reference. This is particularly true for the turn-taking categories, TT(O) and TT(S). Conversely, higher (more positive) values of inhalation slope increase the odds of the reference WSS+INH category. McFadden's pseudo-*R*^2^ of this model equalled 0.06. When duration was added as a predictor, this value changed only slightly to 0.07.

The second model (Table 4), which only includes WSS+INH, TT(O), and TT(S) intervals reflects the same effects for slope and offset level but additionally indicates that increased speech lag increases the odds of the predicted category against the reference. McFadden's pseudo-*R*^2^ of this model equalled 0.21, compared to *R*^2^ of 0.14 for a model with slope and offset level as the only predictors. This relatively high *R*^2^ value compared to the model in Table 3, which included the same predictors, is likely due to the somewhat better separation of the TT(O) and TT(S) categories as well as the reduced number of predicted classes. Addition of inhalation duration to the final model did not substantially improve the model fit (*R*^2^ = 0.22).

### 4.4. Breath Holds in Silent Breathing

**Figure 7:**
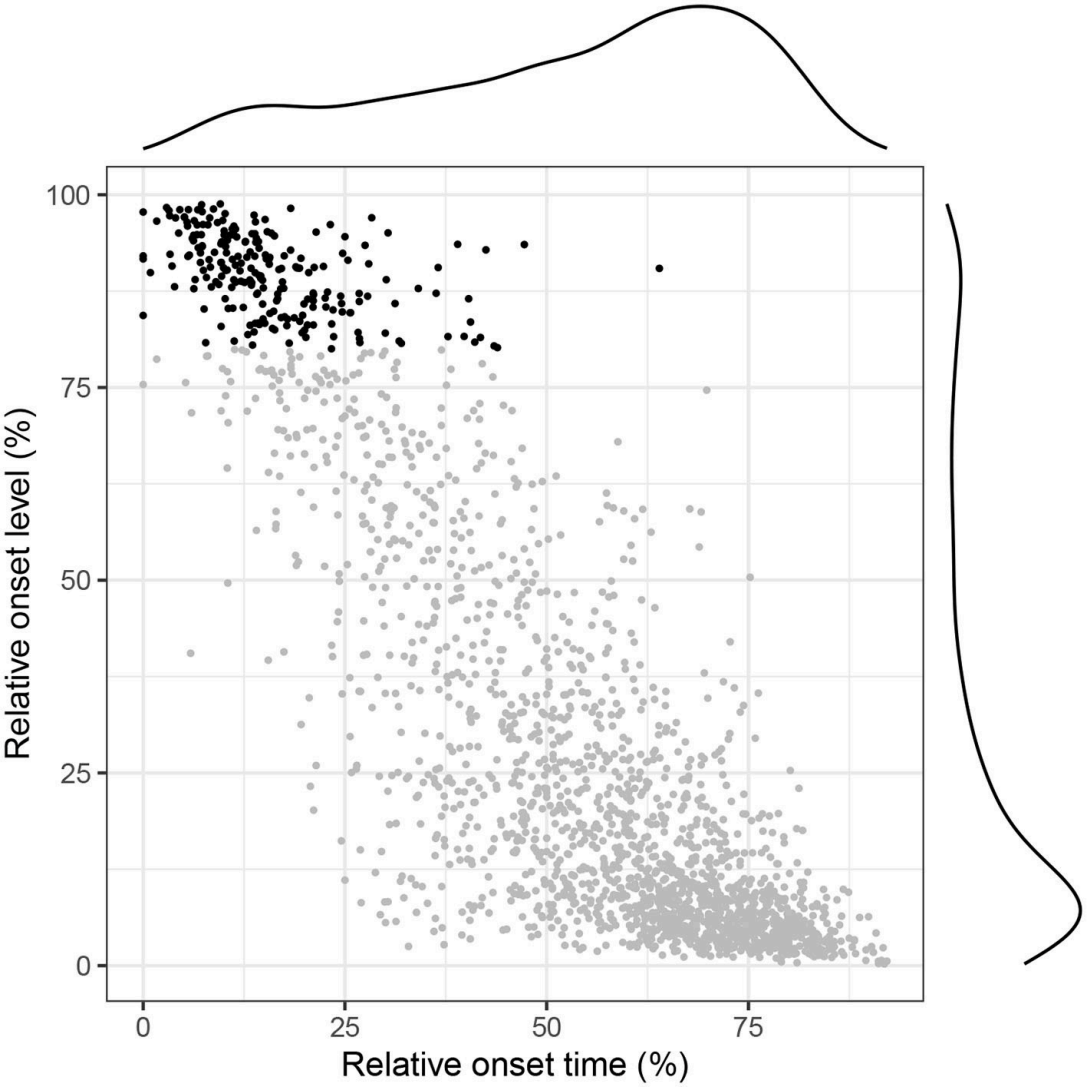
Distribution of holds within the exhalation in silent breathing. Holds occurring in the top 20% of the exhalation amplitude are plotted in black.

In the present section we turn to the problem of identifying abandoned speech initiations. To that end we identified breath holds during silent breathing (i.e., breath holds found in exhalations not coinciding with speech). In Figure 7, we plot their relative position in the exhalation duration (along the abscissa) and the exhalation amplitude (along the ordinate). As is evident both from the scatter plot and the marginal distributions, breath holds occur primarily toward the end (and the bottom) of the exhalation. This grouping is expected given that the silent breathing pattern often exhibits flattened valleys near REL.

**Figure 8:**
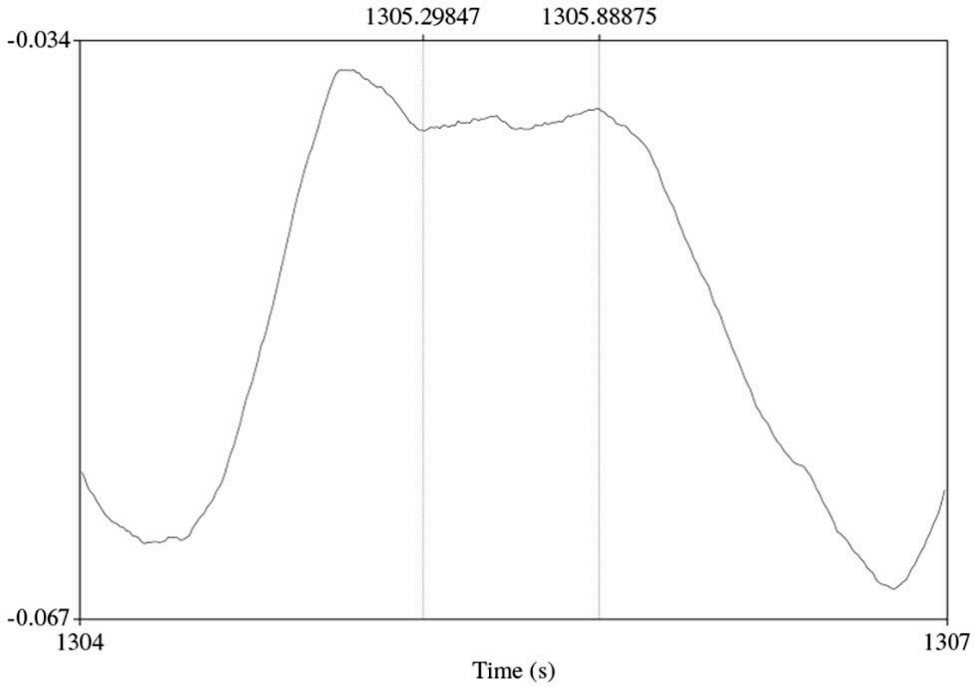
An abandoned initiation candidate: a silent cycle with a respiratory hold (indicated by vertical lines) in the top part of the exhalatory phase.

**Figure 9:**
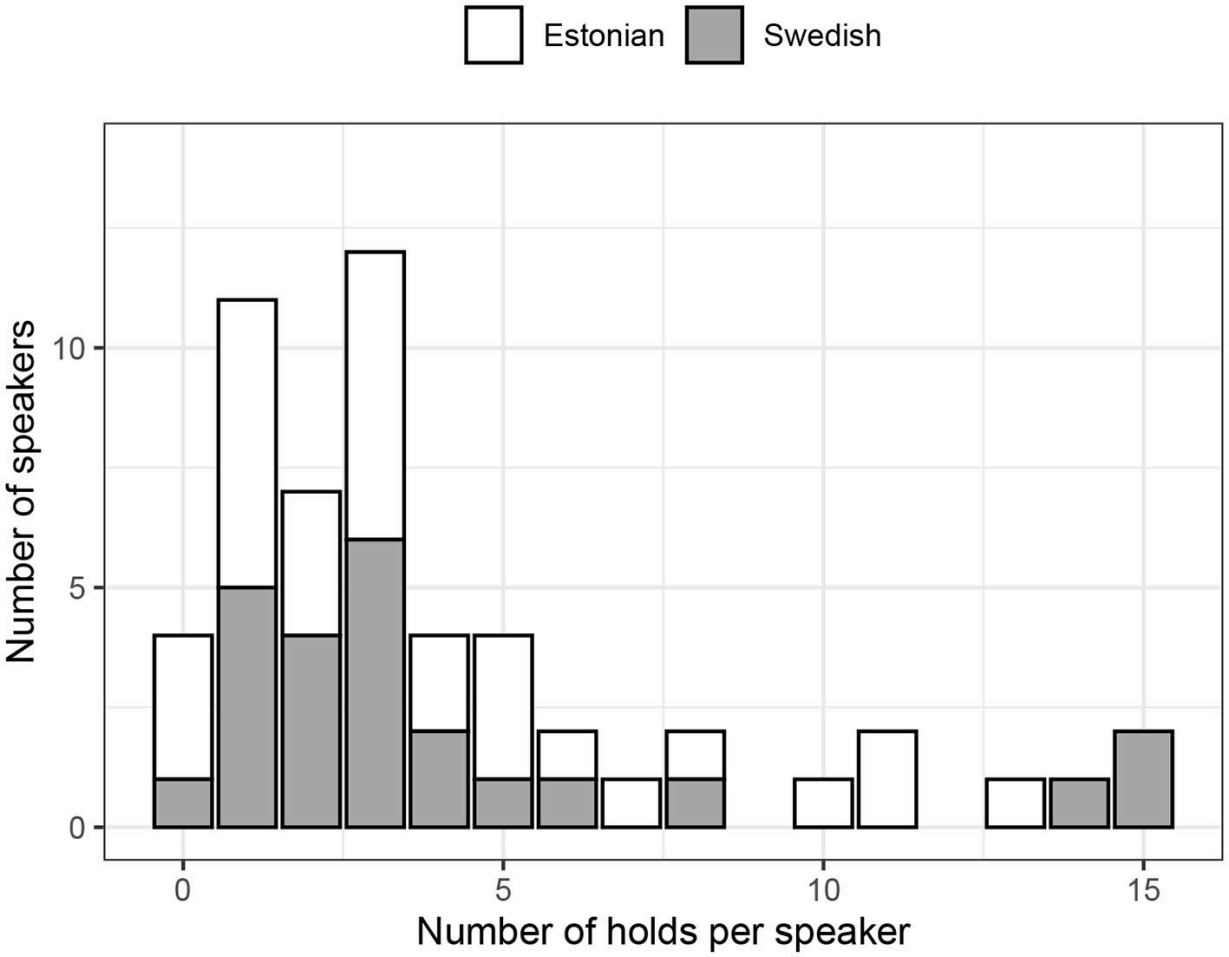
Distribution of silent holds in the top 20% of the exhalation amplitude across speakers and languages. The reader is reminded that each conversation took approximately 20 min.

Of more interest to our present goal is the smaller concentration of respiratory holds toward the beginning (and the top) of the exhalation. This indicates that speakers sometimes hold their breath right after an inhalation. This behavior is illustrated in Figure 8, which clearly shows the speaker holding their breath for about 500 ms right after the inhalation offset and exhaling without producing any speech. The pattern is rather surprising and might indeed suggest that the speaker was getting ready to produce speech but his or her intention was frustrated, for instance because another participant was able to take the turn faster. The frequencies of such silent holds across speakers and languages are shown in Figure 9. Overall, while some speakers seem to produce them more often than others, it is rather uncommon to observe more than five silent holds per speaker and conversation. There were no substantial differences between the two languages.

**Figure 10:**
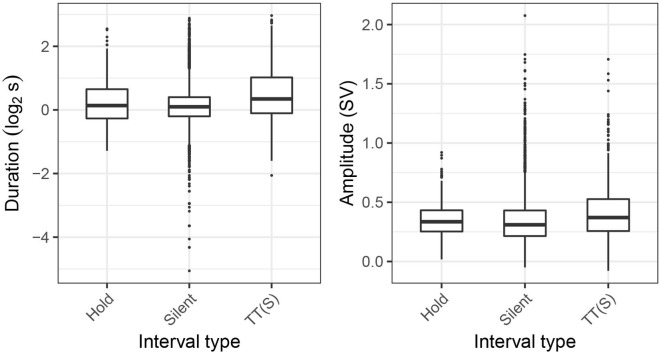
Distribution of inhalation duration and amplitude in silent cycles (Silent), silent cycles coinciding with respiratory holds (Hold), and pre-speech inhalations in TT(S) intervals.

In Figure 10, we plot inhalation durations (in log_2_ s) and inhalation amplitude (as a fraction of the speaker's respiratory range) in silent cycles split depending on whether or not they coincide with a hold, as well as in turn-taking cycles accompanied by silence, TT(S). Overall, the TT(S) category is characterized by both increased inhalation duration and amplitude, with the values for the Hold class placed in between the other two. These tendencies are also reflected in the results of the multinomial logistic regression in Table 5, where increased inhalation duration is associated with a decrease of the odds of a silent cycle and an increase of odds of the TT(S) category. The effect of amplitude was only significant for the TT(S) category, whose odds increase with an increased inhalation depth. However, the differences are very small, which is also reflected in the resulting *R*^2^ values. Even though both inhalation amplitude and slope significantly improve the model fit, McFadden's pseudo-*R*^2^ is quite low (0.03) indicating the predictive power of these features for discrimination between the three categories is limited.

**Table 5:** Coefficients of the inhalatory model for Silent, Hold, and TT(S) intervals.

				**95% CI**		
		**B**	**exp(B)**	**LL**	**UL**	***p***
Silent	Intercept	3.46	31.67	3.18	3.73	0.00
	Amplitude	−0.26	0.77	−0.97	0.45	0.47
	Duration	−0.23	0.80	−0.44	−0.02	0.03
TT(S)	Intercept	0.19	1.21	−0.13	0.51	0.25
	Amplitude	0.94	2.55	0.14	1.73	0.02
	Duration	0.37	1.45	0.12	0.62	0.00

*R^2^ = 0.03 (McFadden), model χ^2^_(6)_ = 144.86, p < 0.01*.

*The reference category is Hold*.

## 5. Discussion and Conclusions

In this paper, we have aimed to provide an overview of respiratory patterns related to several floor management strategies. The basic analytical categories used in the present work were formulated in terms of between- and within-speaker silences and overlaps (Jaffe and Feldstein, 1970). These categories, based entirely on the mechanistic criteria of presence of overlapping speech and speaker change, were further divided into subcategories depending on whether the previous speaker inhaled before producing her next talkspurt. The motivation for these augmented categories was an attempt to uncover pragmatic categories which are normally obscured by purely silence-based representations of interactions, such as pause interruptions or abandoned turn-yields. Here, we assumed that lack of inhalation might be used to mark turn-incompleteness in the previous speaker.

The results summarized in the previous section are largely in line with this assumption. This was particularly true of exhalatory features, which have so far been routinely overlooked in studies of interactional functions of breathing. Specifically, in terms of onset level and slope values, the [−INH] categories all behaved similarly: they were initiated higher in the respiratory range and involved less steep exhalations than the intervals associated with an inhalation, in line with the hypothesized incomplete character of the preceding speech. By contrast, the [+INH] intervals were generally terminated around REL, which is the physiologically motivated point to start an inhalation and (given some degree of linguistic planning) is also the expected endpoint for pragmatically complete breath groups.

At the same time, the BSO-INH and BSS-INH intervals were characterized by the longest exhalations of all the analyzed categories. Given that these intervals, alongside BSO+INH, also had the highest likelihood of coinciding with a respiratory hold, the results are consistent with the idea that these cases involve turn-competition, whereby the previous speaker is trying to keep the turn.

Notably, the WSS-INH intervals were not associated with a high likelihood of a respiratory hold, with speaker changes being more likely in the presence of a respiratory hold [*P*(speaker change|hold) = 0.74] than in its absence [*P*(speaker change|no hold) = 0.38]. This suggests that respiratory holds do not function as a proactive turn-holding resource, as proposed by Local and Kelly (1986). Rather they are employed reactively to maintain possession of or reclaim the conversational floor in the presence of interlocutor's interruption.

By contrast, the analysis of inhalatory features was relatively less revealing. Here, we were mainly able to reproduce the temporal compression effect noted in earlier studies (Rochet-Capellan and Fuchs, 2014; Rochet-Capellan et al., 2014; Ishii et al., 2016), and the associated differences in slope in within-speaker (WSS+INH) intervals. Interestingly, and unlike (Ishii et al., 2016), we found little to no difference between inhalations following between-speaker intervals and those preceding speech in the turn-taking categories. The inhalatory features were also found to have lower predictive power than exhalatory features as quantified by McFadden's pseudo-*R*^2^ of multinomial logistic regression models.

**Figure 11:**
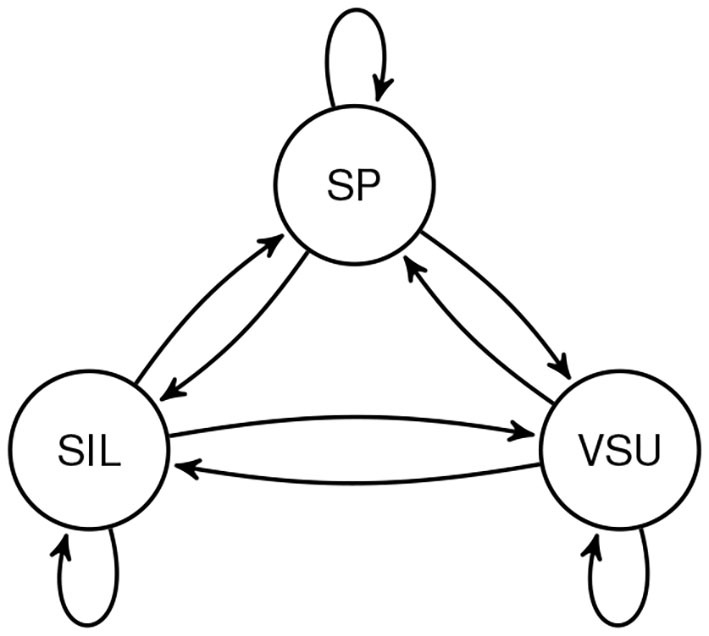
The turn-taking/respiratory model from Włodarczak and Heldner (2016b) showing all possible transitions between respiratory cycle types: speech (SP), very short utterance (VSU), and silent (SIL).

Notably, much of the variation in the exhalatory features would have been completely lost if the respective [+INH] and [−INH] categories had been collapsed. The rather marked grouping visible in Figure 5 suggests, therefore, that our proposed sub-categorization of the interactional events is pragmatically justified. It is also a promising extension of our earlier attempts at combining turn-taking and respiratory categories. We discuss these briefly in the hope that our unsatisfactory solutions will help the reader learn from our mistakes. In Włodarczak and Heldner (2016b), we proposed a simple finite-state model which included all possible transitions between subsequent respiratory cycles of a single speaker depending on whether they coincided with silence, utterances shorter than one second or longer stretches of speech (Figure 11). While this representation allowed us to find some of the interactional effects, such as the time compression in WSS intervals, it was essentially unilateral and did not take any account of the interlocutors' actions. We improved on this deficiency in Włodarczak and Heldner (2018), where exhalations in WSS and BSS intervals were subdivided depending on whether they were followed by an inhalation. Our present approach is thus an extension of that representation to include inhalatory features and intervals with overlapping speech.

Unlike the [±INH] dimension, presence of overlapping speech, which is another routinely overlooked aspect in studies of breathing in turn-taking, played little role discriminating between the categories. With the exception of slightly increased inhalation slope values in TT(O) and BSO+INH in comparison to TT(S) and BSS+INH, the differences were practically nonexistent. In other words, incoming speakers' breathing behavior is not substantially altered by presence of overlapping speech, possibly indicating that the latter is not a reliable indicator of turn competition.

In addition to respiratory patterns in the vicinity of speech, we also investigated occurrences of breath holds in silent cycles with a view to identifying abandoned attempts at taking a turn. Indeed, recent evidence (Aare et al., 2020) suggests that breath holds might be useful for spotting other hidden phenomena such as transitions between chat and chunk phases in casual conversation (Slade, 2007). Overall, the distribution of breath holds exhibited two peaks, toward the beginning and the end of the silent exhalations. This observation is in line with the results reported in Aare et al. (2019), based on manually labeled breath holds in the Estonian subset of the present data. Subsequently, we focused on silent cycles with breath holds early in the exhalation as potential abandoned attempts at taking the turn and compared the properties of the preceding inhalation with those found in other silent cycles and in (realized) turn initiations. While some significant differences were found, the effects were very small and the overall fit of the model poor. At the same time, the difference between the latter two categories was also minor^10^. In addition, we claim the very presence of a respiratory hold coinciding with a silent breathing cycle can be interpreted as a strong indication that the participant was preparing to produce speech. Additionally, if these cycles are used as a signal that an interlocutor is keen to take the conversational floor, it is also possible that they are rendered perceptually prominent by increasing loudness of the inhalation (cf. Włodarczak and Heldner, 2016a, 2017; Trouvain et al., 2020), for instance by producing a narrower constriction in the vocal tract.

In conclusion, the present work has made several non-trivial contributions to its field of study. First, the paper provides a comprehensive overview of respiratory patterns employed in management of casual conversations, including dimensions which have so far been largely overlooked, such as breath holds, exhalations, and presence of overlapping speech. Second, the analyses were based on a larger amount material than used in most previous studies of respiratory turn-taking cues, collected with a custom built RIP system using two belts for estimation of lung volume change. Third, we used a pipeline for automatic analysis of respiratory signals with only minimal manual adjustments. Given that we were able to reproduce many of the earlier findings reported in literature, this suggests that the method used in this study, as well as the RespInPeace toolkit developed by the first author, is a promising way of analysing respiratory RIP signals. Fourth, we proposed an extended classification of interactional events which involves longer sequences than just transitions between interlocutors' adjacent talkspurts and which incorporates respiratory information (presence of inhalation in the previous speaker) for getting closer to competitive/non-competitive speaker changes without the need for an explicit analysis of interlocutors' communicative intentions. Fifth, using these categories, we were able to shed light on *hidden events* in conversations whose identification otherwise requires time-intensive manual analysis of speech content, possibly in connection with other non-verbal cues such as posture shifts, gaze patterns etc.

To the best of our knowledge, this is the first study which attempted to identify unrealized turn-taking intentions using a fully automatic method. By comparing breathing features of hidden event candidates with their overt counterparts (e.g., pause interruptions, i.e., intended pauses within an ongoing turn, with actual within-speaker intervals) we hope to have shown that inclusion of additional data streams can be helpful for this task and we are looking forward to seeing other multimodal data (e.g., gaze) being used for this purpose. At the same time, even though this method provides support to the hypothesized character of the identified events, an independent qualitative investigation of their pragmatic function is certainly warranted, including the question as to whether the identified patterns are actually used by conversation partners for turn-management. Furthermore, analysing patterns of respiratory holds in the vicinity of overlapping speech might be useful for solving the thorny problem of discriminating between collaborative and competitive overlaps (Kurtić et al., 2013; Kurtić and Gorisch, 2018). Notably, prediction and identification of hidden events is crucial for designing of conversational agents capable of human-like turn-taking behavior. Such systems should be able to detect that, for example, the user is about to start speaking or is going to continue their turn after a brief turn-internal silence. The results presented in this work are only a step in this direction. The growing availability of remote breathing sensors, mentioned in section 1, as well as results indicating that visualization of breathing is an important part of embodiment in machine interfaces (Watanabe et al., 2004) suggest that breathing might indeed be helpful for designing truly sociable interaction technology.

In future work, we are planning to investigate prosodic characteristics in the vicinity of the hidden-events identified in this study. In addition, we are conducting an EEG study of preparatory markers of turn-taking (including failed starts), using a hyperscanning paradigm including several conversational partners in parallel. The results will allow evaluating and augmenting the neural evidence for speech planning in conversation (Bögels et al., 2015), which has been based so far on partly controlled experiments, in a fully interactive context.

## Data Availability Statement

The datasets presented in this study can be found in online repositories. The names of the repository/repositories and accession number(s) can be found at: https://doi.org/10.5281/zenodo.4054803.

## Ethics Statement

Ethical review and approval was not required for the study on human participants in accordance with the local legislation and institutional requirements. The patients/participants provided their written informed consent to participate in this study. Written informed consent was obtained from the individual(s) for the publication of any potentially identifiable images or data included in this article.

## Author Contributions

MW and MH: conception and design of the work, acquisition, analysis, and interpretation of the data, drafting the work, and final approval of the version to be published. All authors contributed to the article and approved the submitted version.

## Conflict of Interest

The authors declare that the research was conducted in the absence of any commercial or financial relationships that could be construed as a potential conflict of interest.

## Footnotes

^1^Notably, the interruptive character of incoming speech was independent of presence of overlap. Thus, pause interruptions rand interruptions accompanied by overlap were not distinguished.

^2^Existing studies use threshold values ranging widely from 50 (Gravano and Hirschberg, 2011) to 500 ms (Shriberg et al., 2001), with the perceptual threshold on pause perception estimated at around 120 ms (Heldner, 2011).

^3^https://github.com/jrgillick/laughter-detection (accessed August 13, 2019).

^4^A manual resegmentation of the Estonian data set (not used in this work) resulted in adjustment of 12.6% of the automatic boundaries. Since no manual segmentation was available for the Swedish data, we chose to use the automatic method for the sake of consistency.

^5^This is less of a problem for WSS-INH since in those cases the next talkspurt of the previous speaker coincides with the end of the silent interval. By contrast, for BSO-INH and BSS-INH intervals the next talkspurt of the previous speaker might not occur until much later in the conversation.

^6^We feel that the relatively high number of excluded cases can be attributed primarily to inaccuracies in breath segmentation (although see footnote 4 for a comparison with a manual resegmentation of a subset of the data) and the low time resolution (100 ms) in the voice activity detection used, which increased the chances for a speech segment to spill over into the inhalation on either side.

^7^Note that speech lag is undefined for BSS+INH since in those cases the following breathing cycle of the previous speaker need not include a speech segment.

^8^Admittedly, the 20% cutoff was quite arbitrary and was chosen as a trade-off between selecting the most likely abandoned speech candidates, located toward the top of the exhalation range, and ensuring an acceptable sample size.

^9^The reader is reminded that presence of correlated predictors adversely affects parameter estimates and their associated *p*-values but not the overall model fit (Harrell, 2001).

^10^Somewhat surprisingly, unlike Ishii et al. (2016), in this work we were unable to find evidence for substantial differences in inhalation amplitude between the analyzed categories. Whether this might be an result of the character of our data or the analysis method used is unclear. It should also be noted that Ishii et al. (2016) used only a single (abdomen) belt and consequently their volume-related estimates might be unreliable.
